# Less invasive corrective surgery using oblique lateral interbody fusion (OLIF) including L5-S1 fusion for severe lumbar kyphoscoliosis due to L4 compression fracture in a patient with Parkinson’s disease: a case report

**DOI:** 10.1186/s13104-015-1087-y

**Published:** 2015-04-07

**Authors:** Hiromasa Wakita, Yasuhiro Shiga, Seiji Ohtori, Go Kubota, Kazuhide Inage, Takeshi Sainoh, Jun Sato, Kazuki Fujimoto, Kazuyo Yamauchi, Junichi Nakamura, Kazuhisa Takahashi, Tomoaki Toyone, Yasuchika Aoki, Gen Inoue, Masayuki Miyagi, Sumihisa Orita

**Affiliations:** Department of Orthopaedic Surgery, Graduate School of Medicine, Chiba University, 1-8-1 Inohana, Chuo-ku, Chiba 260-8670 Japan; Department of Orthopaedic Surgery, Teikyo University Mizonokuchi Hospital, Tokyo, Japan; Department of Orthopaedic Surgery, East Chiba Medical Center, Chiba, Japan; Department of Orthopaedic Surgery, Kitasato University, Kanagawa, Japan

**Keywords:** Kyphoplasty, Minimally invasive corrective surgery, Oblique lateral lumbar interbody fusion (OLIF)

## Abstract

**Background:**

Corrective surgery for kyphoscoliosis patients tend to be highly invasive due to osteotomy. The present case introduce less invasive corrective surgery using anterior oblique lateral interbody fusion (OLIF) technique.

**Case presentation:**

An 80-year-old Japanese man with a history of Parkinson’s disease presented to our hospital because of severe kyphoscoliosis and gait disturbance. Considering the postsurgical complications due to osteotomy, we performed an anterior-posterior combined corrective fusion surgery: OLIF of Lumbar (L) 2-3, L3-4, and L4-5 (Medtronic Sofamor Danek, Memphis, TN, USA) followed by L5-Sacral (S) 1 anterior lumbar fusion via the OLIF approach using an anterior intervertebral cage, and posterior L3-4 and L4-5 facetectomy and posterior fusion using percutaneous pedicle screws from Thoracic (T) 10 to S1 with a T-9 hook system. The surgery was performed in a less invasive manner with no osteotomy, and it improved the sagittal alignments with moderate restoration, which improved the patient’s posture and gait disturbance. The patient showed transient muscle weakness of proximal lower extremity contralateral side to the surgical site, which fully recovered by physical rehabilitation 3 months after the surgery.

**Conclusion:**

The surgical corrective procedure using the minimally invasive OLIF method including L5-S1 fusion showed a great advantage in treating degenerative kyphoscoliosis in a Parkinson’s disease patient in its less-invasive approac.

## Background

Spinal degenerative kyphoscoliosis is currently a major locomotive problem. Adult degenerative scoliosis has an estimated prevalence of 6% in people over 50 years of age [1]. Patients with this disorder may present gait disturbance, gastroesophageal reflux disease (GERD), back pain, leg pain, and neural deficit. Patients with spinal deformity derived from Parkinson’s disease (PD) present an abnormal posture of the trunk with marked flexion of the thoracolumbar spine, which is known as bent spine syndrome or camptocormia [2]. Camptocormia patients sometimes need corrective surgery to maintain their activities of daily life or quality of life by correcting or straightening their posture. Spinal fusion surgery in PD patients tends to cause non-union and implant failure due to PD; thus, a more rigid and corrective surgical procedure is needed, such as vertebral column resection (VCR) or pedicle subtraction osteotomy (PSO). These procedures include osteotomy and long posterior fusion, which may also cause postsurgical complications such as non-bony union and implant failure. The aim of the present report is to describe our novel surgical procedure for addressing these deficits.

## Case presentation

**Figure 1:**
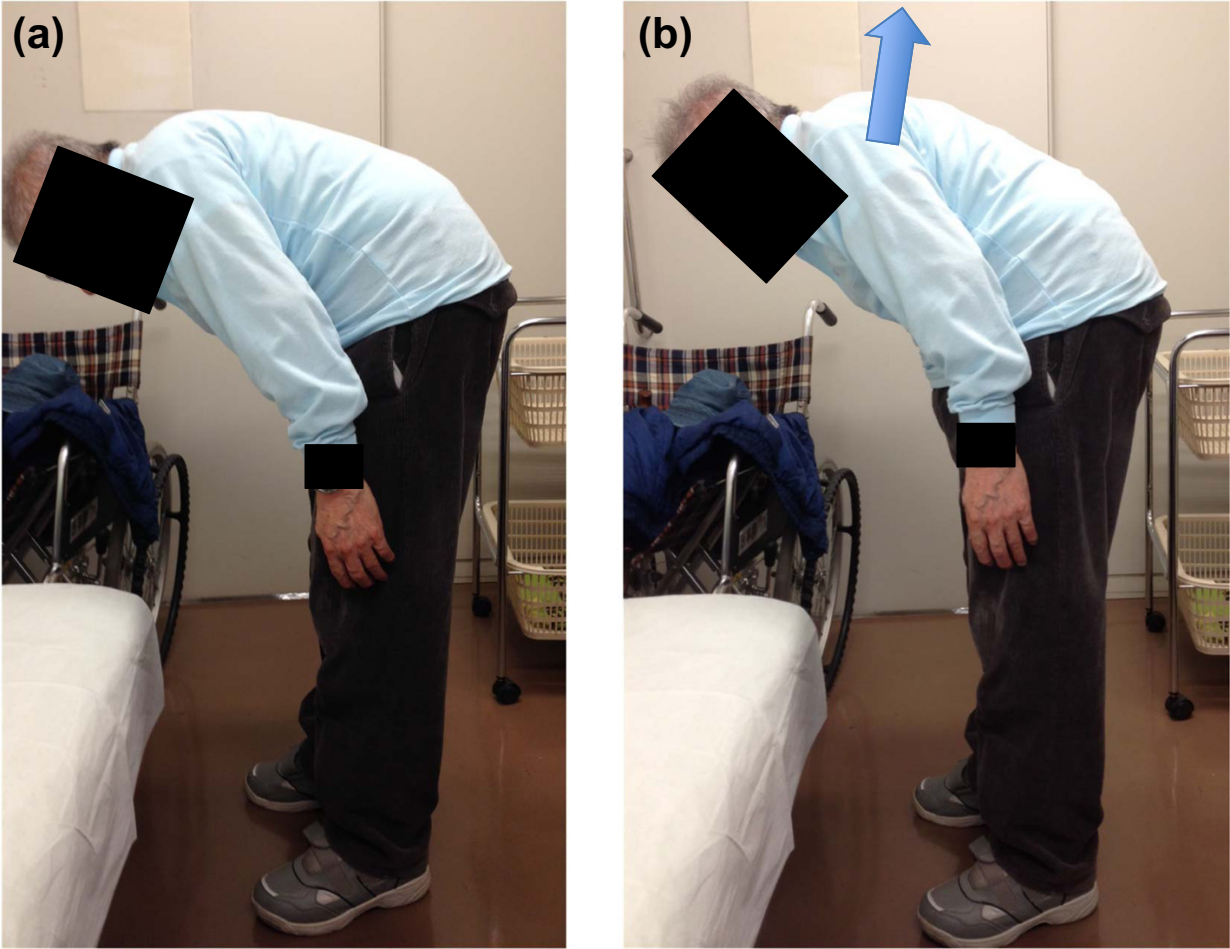
**The patient showed severe kyphosis. (a)** Natural standing position **(b)** Extension position with maximum effort. Note that the patient is not able to gaze straight forward even at maximum extension.

An 80-year-old Japanese man was referred to our outpatient clinic because of severe kyphoscoliosis and gait disturbance. Ten years previously, he had been diagnosed with PD with a tendency to adopt a flexion position, which had gradually exacerbated in the last four years, resulting in severe kyphoscoliosis. He complained of difficulty maintaining a neutral position and inability to gaze straight forward, which led to gait disturbance in addition to that from PD (Figure 1). He was only able to walk for short periods, using a gait trainer, because of fatigue from his posture. He complained of another symptom of occasional reflux or heartburn from GERD.

**Figure 2:**
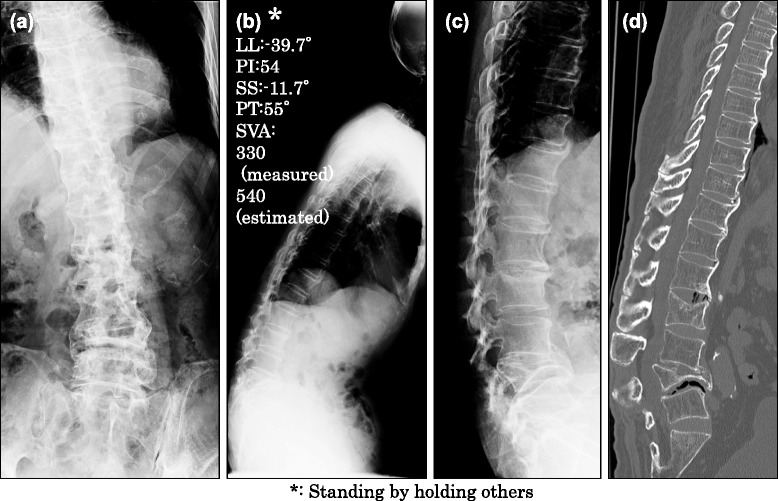
**Plain radiograph showed rigid kyphoscoliosis. (a)** The antero-posterior view showed slight left-convex scoliosis with deformity. **(b)** Whole spinal lateral view. Note that the patient was only able to maintain the standing position with assistance; thus, the image is not a true whole-spinal image. **(c)** The lateral view at maximum extension hardly showed changes in alignment. **(d)** Computed tomography scan sagittal image. LL: lumbar lordosis, PI: pelvic incidence, SS: sacral slope, PT: pelvic tilt, SVA: sagittal vertical axis.

The patient’s physical examination revealed extremely decreased range of motion (ROM) in lumbar flexion and extension with no neurological disorder such as motor function loss and sensory disturbance. He presented limited hip extension because of the rigid long-term kyphoscoliosis. Radiographic findings of his thoracolumbar spine showed severe rigid kyphosis due to a Lumbar (L) 4 burst fracture (semiquantitative grade 3) with little ROM in extension/flexion (Figure 2a-c) with no episodes of fall or trauma that may have caused the L4 fracture. His bone mineral density was within a normal range with no evidence of osteoporosis. Sagittal alignment parameters were measured as: lumbar lordosis (LL) -39.7°, pelvic incidence (PI) 54°, sacral slope (SS) -11.7°, pelvic tilt (PT) 55°, and sagittal vertical axis (SVA) 330 mm. Regarding the SVA, the measured value was not true because the patient was unable to stand alone without assistance, and 540 mm was the estimated value by considering his physique. A computed tomography scan and magnetic resonance imaging showed invagination of the fractured posterior wall of the L4 vertebrae without harmful interruption in the spinal canal or foramen. The intervertebral discs of L1-2 and L4-5 showed degeneration with vacuum phenomenon (Figure 2d).

In planning the surgical method, we calculated the ideal restoration for the LL as 25–45° using Schwab’s formula, [3] requiring about 65–80° of restoration. However, we decided not to perform any osteotomy such as VCR or PSO to acquire that LL restoration, considering the PD-related postsurgical complications such as delay in bony union and implant failure [2]. Thus, we performed anterior-posterior combined corrective fusion surgery: anterior oblique lateral lumbar fusion (OLIF) of L2-3, L3-4, and L4-5 (using a Clydesdale cage, Medtronic Sofamor Danek, Memphis, TN, USA) followed by L5-Sacral (S) S1 anterior lumbar fusion via the OLIF approach using an anterior intervertebral cage (SynCage®, Depuy Synthes, Raynham, MA, USA), and posterior L3-4 and L4-5 facetectomy and posterior fusion using percutaneous pedicle screws from Thoracic (T) 10 to S1 with a T-9 hook system (Medtronic).

**Figure 3:**
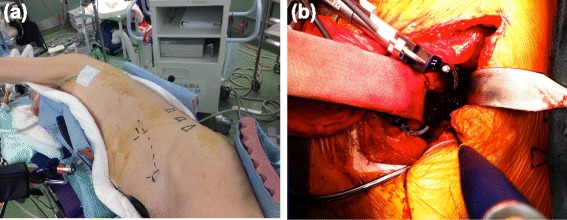
**Intraoperative photograph. (a)** skin incision was made 12 cm anterior from the mid portion of the L5-S1 disc. **(b)** The oblique lateral lumbar fusion retractor system was used to approach the lumbar intervertebral discs from L2-3 to L5-S1 via the retroperitoneal space.

**Figure 4:**
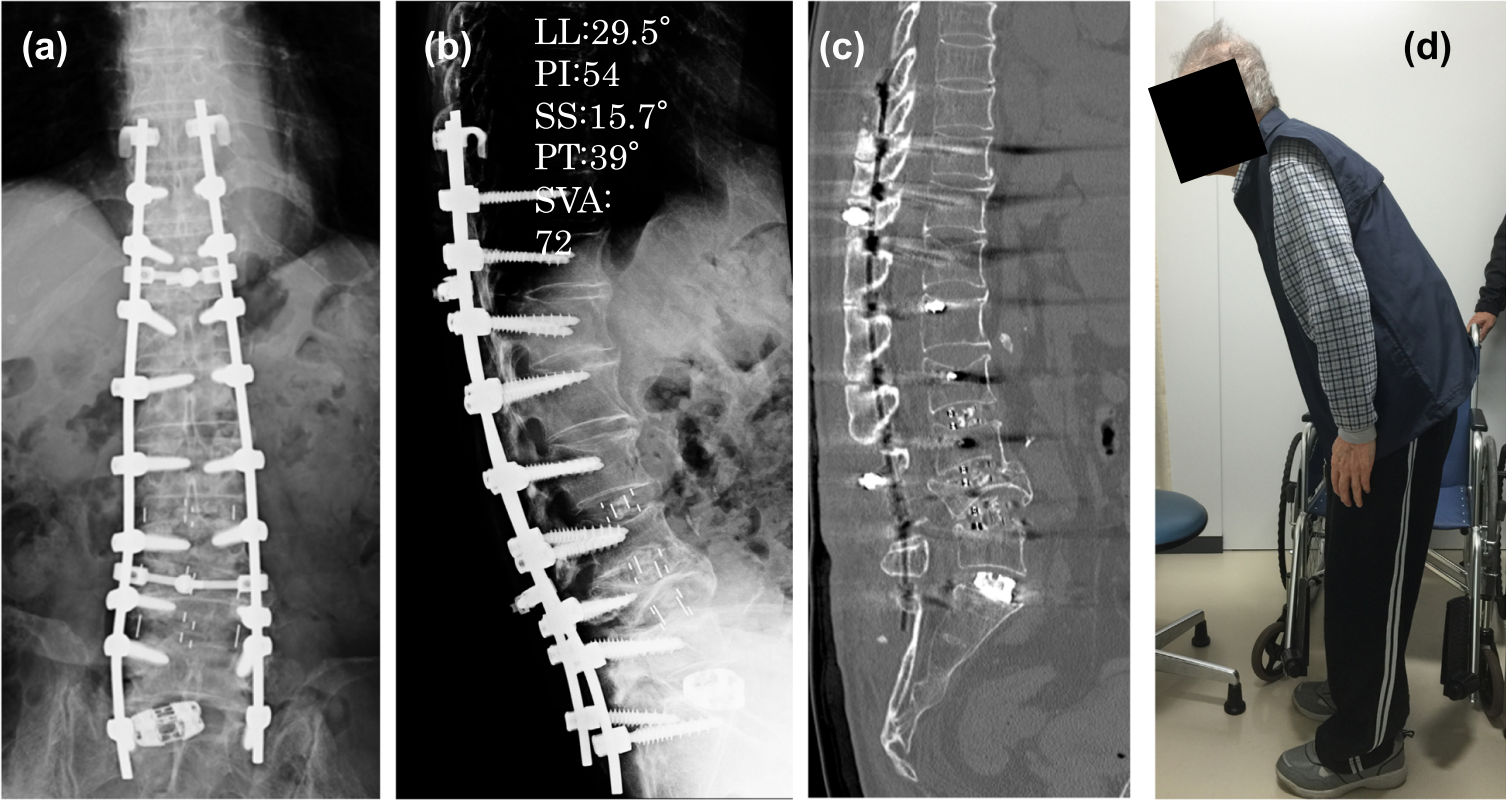
**Postsurgical images. (a)** Antero-posterior view. **(b)** Lateral view. **(c)** Computed tomography scan lateral image. **(d)** Maximum extension position. The patient can stand alone gazing straight forward.

In detail, the patient was set in a prone position and underwent posterior fusion with L3-4 and L4-5 facetectomy first, to acquire mobility in the posterior part of the lower back. Pedicle screws were inserted transfascially at T10-L2 and L5-S1. The L3-5 lamina was exposed in the usual manner, and L3-4 and L4-5 facetectomy were performed. Then, the patient was set in a lateral position on his right. After the confirmation of L2-S vertebrae/sacrum, a 12-cm skin incision was made about 12 cm anterior to the marked L5-S1 disc space (Figure 3a). Regarding L2-3, L3-4, and L4-5, Crydesdale® cages were inserted using an OLIF retractor (L2-3: 10-mm height × 55-mm long; L3-4 and L4-5: 14-mm height × 55-mm long). After the OLIF procedure, the bifurcations of the aorta and vena cava were exposed for the following L5-S1 access, and then an anterior L5-S1 cage was inserted according to the previous procedure (Figure 3b) [4]. After the anterior fusion, the patient was set in the prone position again, and the pedicle screws were fixed with rods with compression at L3-5. The total surgical time was 5:07 (hr:min. first posterior, 1:37; anterolateral, 3:05; and second posterior, 1:25), and the intrasurgical blood loss was 360 g in total. Postsurgery, the patient was able to stand alone and still, and to gaze straight forward (Figure 4d). He experienced transient decrease in proximal muscle strength in his lower limb of contralateral side of the surgery (manual muscle test (MMT) grade 2 of 5 in iliopsoas and quadriceps muscles), which fully recovered to MMT grade 5 within 3 months after the surgery by daily muscle strengthening exercise and gait. In detail, He required a support of walker to stand still till the end of the 1 month after the surgery, and gradually acquired the ability of hip flexion. Once he acquired the proximal muscle power, he came to walk by himself day-by-day about 2.5 months after the surgery under the supervision of physical trainers. The transient muscle weakness was thought to be from some anatomical change in the nerves such as the corrected sagittal alignment. His presurgical symptoms of GERD have disappeared, and his SVA has improved from an estimated 540 mm to 212 mm just after the surgery, which is decreasing gradually together with the improving sagittal alignment and hip ROM. His LL, PT, and SS have improved to 29.5°, 39°, and 15.7°, respectively. Regardless of the non-fully restored postsurgical lordosis and SVA, the patient was satisfied with the surgical outcome, as he can now walk with only a cane, gazing straight forward for at least 30 minutes.

## Discussion

Corrective surgery for degenerative kyphoscoliosis in PD patients is sometimes challenging. PD tends to cause frequent post-surgical complications such as implant failure from motor fluctuations and freezing, which can cause severe instability leading to massive implant failure even in a good PD control. A disorder of the involved muscle such as the abdominal and paraveretebral muscle is considered to be the cause of the issue [5].

Generally, kyphoscoliosis can be treated with osteotomy surgeries such as VCR or PSO to acquire enough LL and SVA restoration, including a previously reported method of asymmetrical PSO for 3-dimensional spinal correction [6]. However, these procedures with osteotomy can cause severe complications such as instability or implant failure because of their aggressive surgical invasion. In addition, synergistically, PD can cause a high incidence of postsurgical complications. The present case showed an L4 burst fracture with no episode of fall and no evidence of osteoporosis, which may have been induced by the PD-related camptocormia, suggesting strong flexion stress on the lower back. The fracture can lead to possible collapse at the anterior site of the osteotomy in VCR or PSO, which was of great concern in the present case. One of the issues with osteotomy in the present case is that PD patients have difficulty in achieving bony union and stabilization because of the micromotion and unpredic muscle tension.

In correcting sagittal malalignment, posterior instrumented fusion alone is not enough because the procedure tends to restore the deformity with focus on the coronal rather than the sagittal plane. Some studies have reported on the effect of lateral lumbar interbody fusion for correcting spinal malalignment [7-10]. Lateral interbody fusion (LIF) has been reported recently as a less-invasive corrective surgery, which enables significant gain in the segmental LL [9] and restoration of the segmental disc height, coronal angle, and lordotic angle [7]. Thus, multilevel LIF is useful in corrective surgery to acquire global sagittal alignment by accumulating the restored angle in LL followed by SVA improvement. Mostly performed recent LIF procedure is the transpsoas extreme lateral interbody fusion (XLIF) [11-13], mainly because XLIF was introduced 4–5 years before OLIF. OLIF and XLIF have the same concept of LIF, but with different approaches. OLIF is a mini-open retroperitoneal surgery, which enables a relatively easier additional approach to the L5-S1 junction than XLIF. One of the strong points in the present case is the ability to perform L5-S1 fusion via the same incision of OLIF, which we have previously reported [4].

There were some limitations and problems in the present case. First, full restoration of the LL was not achieved. According to Schwab’s formula, the ideal value was calculated as 25–45°, which is known as the standard sagittal alignment correction value [3]. We may have achieved the ideal LL value with procedures involving osteotomy; however, there was a high possibility of complications, as described above. In the present case, more posterior facetectomy and higher intervertebral cages may be needed to achieve full restoration. The insufficient restoration of the sagittal alignment may also include the contraction of the hip joint due to the long-term thoracolumbar kyphoscoliosis, while he shows gradual improvement in his posture, indicating the possibility of further restoration in his sagittal alignment. Second, the patient showed transient postsurgical proximal motor dysfunction in the lower limbs. LIF is known to cause transient postsurgical neural disorder due to traction or direct invasion to the intramuscular nerve plexus in the psoas muscle [14]. In addition, the restoration of anterior disc height may have caused this by excess tension to the spinal nerves.

## Conclusion

The surgical corrective procedure using the minimally invasive OLIF method including L5-S1 fusion showed a great advantage in treating degenerative kyphoscoliosis in a PD patient in its less-invasive approach. A more effective procedure to acquire enough restoration of sagittal alignment, including lumbar lordosis, should be developed.

## Consent

Written informed consent was obtained from the patient for publication of this Case Report and any accompanying images. A copy of the written consent is available for review by the Editor-in-Chief of this journal.

## Abbreviations

OLIF: Oblique lateral interbody fusion
T: Thoracic spine
L: Lumbar spine
S: Sacrum
PD: Parkinsons disease
GERD: Gastroesophageal reflux disease
VCR: Vertebral column resection
PSO: Pedicle subtraction osteotomy
LL: Lumbar lordosis
PI: Pelvic incidence
SS: Sacral slope
PT: Pelvic tilt
SVA: Sagittal vertical axis
XLIF: eXtreme lateral interbody fusion

## References

[1] Amin BY, Mummaneni PV, Ibrahim T, Zouzias A, Uribe J (2013). Four-level minimally invasive lateral interbody fusion for treatment of degenerative scoliosis. Neurosurg Focus.

[2] Wadia PM, Tan G, Munhoz RP, Fox SH, Lewis SJ, Lang AE (2011). Surgical correction of kyphosis in patients with camptocormia due to Parkinson’s disease: a retrospective evaluation. J Neurol Neurosurg Psychiatry..

[3] Schwab F, Patel A, Ungar B, Farcy JP, Lafage V (2010). Adult spinal deformity-postoperative standing imbalance: how much can you tolerate? An overview of key parameters in assessing alignment and planning corrective surgery. Spine..

[4] Kanno K, Ohtori S, Orita S, Yamauchi K, Eguchi Y, Aoki Y, et al. Miniopen Oblique Lateral L5-S1 Interbody Fusion: A Report of 2 Cases. Case Reports Orthopedics 2014;603531 10.1155/2014/603531 PMC4221972 25400963

[5] Melamed E, Djaldetti R (2006). Camptocormia in Parkinson’s disease. J Neurol..

[6] Toyone T, Shiboi R, Ozawa T, Inada K, Shirahata T, Kamikawa K (2012). Asymmetrical pedicle subtraction osteotomy for rigid degenerative lumbar kyphoscoliosis. Spine..

[7] Kotwal S, Kawaguchi S, Lebl D, Hughes A, Huang R, Sama A, Cammisa F, Girardi F: Minimally Invasive Lateral Lumbar Interbody Fusion: Clinical and Radiographic Outcome at a Minimum 2-year Follow-up. J Spinal Disorders Tech. 2012. [Epub ahead of print]. http://www.ncbi.nlm.nih.gov/pubmed/?term=Minimally+Invasive+Lateral+Lumbar+Interbody+Fusion%3A+Clinical+and+Radiographic+Outcome+at+a+Minimum+2-year+Follow-up. 10.1097/BSD.0b013e3182706ce7 22964885

[8] Mundis GM, Akbarnia BA, Phillips FM (2010). Adult deformity correction through minimally invasive lateral approach techniques. Spine..

[9] Yson SC, Sembrano JN, Santos ER, Luna JT, Polly DW (2014). Does prone repositioning before posterior fixation produce greater lordosis in lateral lumbar interbody fusion (LLIF)?. J Spinal Disord Tech..

[10] Johnson RD, Valore A, Villaminar A, Comisso M, Balsano M (2013). Pelvic parameters of sagittal balance in extreme lateral interbody fusion for degenerative lumbar disc disease. J Clin Neurosci Off J Neurosurg Soc Australasia..

[11] Malham GM, Ellis NJ, Parker RM, Blecher CM, White R, Goss B, Seex KA: Maintenance of Segmental Lordosis and Disc Height in Standalone and Instrumented Extreme Lateral Interbody Fusion (XLIF). J Spinal Disorders Tech. 2014. [Epub ahead of print]. http://www.ncbi.nlm.nih.gov/pubmed/?term=Maintenance+of+Segmental+Lordosis+and+Disc+Height+in+Standalone+and+Instrumented+Extreme+Lateral+Interbody+Fusion+(XLIF). 10.1097/BSD.0b013e3182aa4c94 28207620

[12] Phillips FM, Isaacs RE, Rodgers WB, Khajavi K, Tohmeh AG, Deviren V (2013). Adult degenerative scoliosis treated with XLIF: clinical and radiographical results of a prospective multicenter study with 24-month follow-up. Spine..

[13] Berjano P, Lamartina C (2013). Far lateral approaches (XLIF) in adult scoliosis. European Spine J Off Publ European Spine Soc European Spinal Deformity Soc European Section Cervical Spine Res Soc..

[14] Grimm BD, Leas DP, Poletti SC, Johnson DR, 2nd: Postoperative Complications Within the First Year After Extreme Lateral Interbody Fusion: Experience of the First 108 Patients. J Spinal Disorders Tech. 2014. [Epub ahead of print]. http://www.ncbi.nlm.nih.gov/pubmed/?term=Postoperative+Complications+Within+the+First+Year+After+Extreme+Lateral+Interbody+Fusion%3A+Experience+of+the+First+108+Patients. 10.1097/BSD.0000000000000121 27007791

